# Contribution of Anoctamins to Cell Survival and Cell Death

**DOI:** 10.3390/cancers11030382

**Published:** 2019-03-19

**Authors:** Karl Kunzelmann, Jiraporn Ousingsawat, Roberta Benedetto, Ines Cabrita, Rainer Schreiber

**Affiliations:** Institut für Physiologie, Universität Regensburg, Universitätsstraße 31, D-93053 Regensburg, Germany; Jiraporn.Ousingsawat@vkl.uni-regensburg.de (J.O.); Roberta.Benedetto@vkl.uni-regensburg.de (R.B.); Ines.Cabrita@vkl.uni-regensburg.de (I.C.); Rainer.Schreiber@vkl.uni-regensburg.de (R.S.)

**Keywords:** anoctamin, ANO1, ANO6, TMEM16A, TMEM16F, cancer, proliferation, apoptosis, Ca^2+^ signaling, inflammation

## Abstract

Before anoctamins (TMEM16 proteins) were identified as a family of Ca^2+^-activated chloride channels and phospholipid scramblases, the founding member anoctamin 1 (ANO1, TMEM16A) was known as DOG1, a marker protein for gastrointestinal stromal tumors (GIST). Meanwhile, ANO1 has been examined in more detail, and the role of ANO1 in cell proliferation and the development of different types of malignomas is now well established. While ANO5, ANO7, and ANO9 may also be relevant for growth of cancers, evidence has been provided for a role of ANO6 (TMEM16F) in regulated cell death. The cellular mechanisms by which anoctamins control cell proliferation and cell death, respectively, are just emerging; however, the pronounced effects of anoctamins on intracellular Ca^2+^ levels are likely to play a significant role. Recent results suggest that some anoctamins control membrane exocytosis by setting Ca^2+^_i_ levels near the plasma membrane, and/or by controlling the intracellular Cl^−^ concentration. Exocytosis and increased membrane trafficking induced by ANO1 and ANO6 may enhance membrane expression of other chloride channels, such as CFTR and volume activated chloride channels (VRAC). Notably, ANO6-induced phospholipid scrambling with exposure of phosphatidylserine is pivotal for the sheddase function of disintegrin and metalloproteinase (ADAM). This may support cell death and tumorigenic activity of IL-6 by inducing IL-6 trans-signaling. The reported anticancer effects of the anthelminthic drug niclosamide are probably related to the potent inhibitory effect on ANO1, apart from inducing cell cycle arrest through the Let-7d/CDC34 axis. On the contrary, pronounced activation of ANO6 due to a large increase in intracellular calcium, activation of phospholipase A2 or lipid peroxidation, can lead to ferroptotic death of cancer cells. It therefore appears reasonable to search for both inhibitors and potent activators of TMEM16 in order to interfere with cancer growth and metastasis.

## 1. Introduction

Cl^−^ currents activated by an increase in intracellular Ca^2+^ (CaCC) have been known for more than 40 years. The human homologue of *Drosophila* tweety and the bestrophin family of channels were shown to operate as Ca^2+^ activated Cl^−^ channels (reviewed in [1,2,3]). However, they behave differently from the “classical” receptor-operated CaCC, identified 11 years ago as anoctamin 1 (ANO1; TMEM16A) [4,5,6]. ANO1 is particularly expressed in acinar cells of secretory glands and is regulated by CLCA1 [7,8]. Apart from glands, CaCCs have long been known to be present primarily in proliferating cells in culture and various types of cancer cells [9,10,11]. After identification of ANO1 as Ca^2+^ activated Cl^−^ channel, it became clear that the protein is identical to DOG1, a significant and reliable tumor marker in gastrointestinal stromal tumors (GIST) and head and neck cancers [12,13,14] (Table 1). Meanwhile, ANO1 has been found in a number of different malignant tumors. Apart from ANO1, other members of the anoctamin family were also correlated with cell proliferation and cancer development, like ANO5 (TMEM16E), ANO7 (TMEM16G) and ANO9 (TMEM16J) (Table 1). Anoctamins could have tumor-specific functions, or may support cell proliferation and possible development towards malignancy in any cell-type. The latter assumption is supported by the fact that ANO1 is present in many different types of proliferating cells and tumor tissues [15] (Table 1). Notably, the ANO1-knockout mouse is hypotrophic when compared to wild type littermates [16]. ANO1 and its role in proliferation and cancer development has been reported repeatedly, but we are still far from any comprehensive understanding. Compared to Ano1, much less is known for other anoctamin paralogues regarding their potential role in proliferation and tumor development (Table 1). Moreover, some anoctamins, like ANO6, may even promote cell death, rather than growth.

## 2. Anoctamins and Their Cellular Localization

Anoctamins form a family of Ca^2+^-activated proteins, consisting of phospholipid scramblases and ion channels [90,91]. The 10 proteins (ANO1-10; TMEM16A-K) are broadly expressed in epithelial and non-epithelia tissues [15]. ANO1 appears to operate as a relatively selective anion channel [92], while ANO6 is a phospholipid scramblase, i.e., it moves phosphatidylserine from the inner to the outer plasma membrane leaflet, when activated by a large increase in intracellular Ca^2+^ [93,94]. However, ANO6 is also permeable for chloride ions [95,96,97]. Previous work suggests that it becomes increasingly nonselective with increasing concentrations of intracellular free Ca^2+^ [98]. Although it is now clear that most anoctamins operate as phospholipid scramblases [99,100,101], our earlier work may suggest that all anoctamins also conduct ions, when co-expressed with purinergic receptors and activated by stimulation with ATP [102]. A subsequent study on the role of ANO5 for muscle repair presented strong evidence that ANO5 is a scramblase and conducts ions as well [103]. 

**Figure 1:**
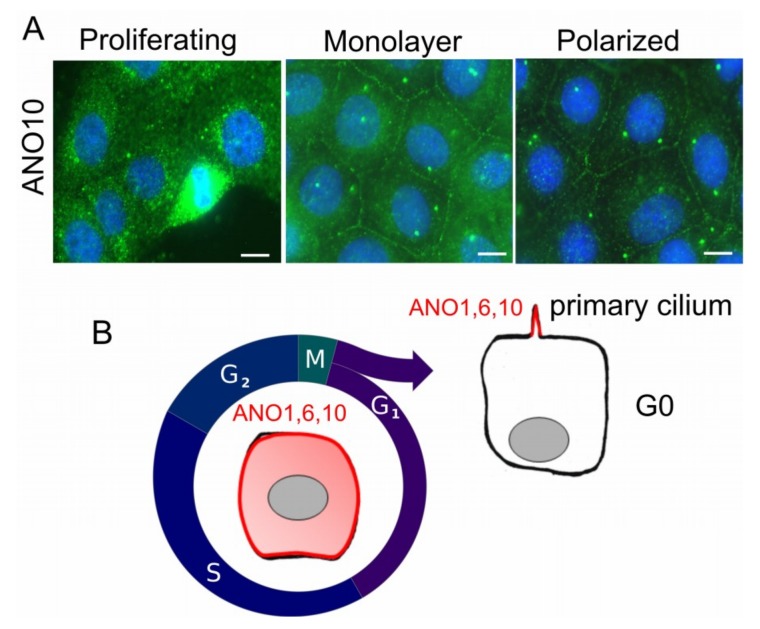
Proliferation-dependent expression of ANO10 in FRT cells. (**A**) FRT cells were grown in FCS-containing media at 70% density (Proliferating), as confluent monolayer in FCS-free media (Monolayer), or as polarized monolayer on permeable supports and in FCS-free media (Polarized). Expression of endogenous ANO10 (green fluorescence) was intracellular in dividing cells (Proliferating), but was detected in the plasma membrane and in the primary cilium in densely grown cells (Monolayer). ANO10 was more prominent in plasma membrane and primary cilium in polarized cells (Polarized). For further details and references, see main text. (**B**) Hypothetical model proposing variable cellular locations of ANO10 depending on cell proliferation or cell polarization. ANO10 is found primarily intracellularly, but is also in the plasma membrane during cell cycle. Reduced expression of ANO10 and translocation into the primary cilium is observed once cells move into G_0_. Bar, 20 µm [108].

It is not entirely clear to what extent anoctamins operate as channels/scramblases in the apical plasma membrane of polarized cells, and what fraction of the protein resides in intracellular membranous compartments, or in the basolateral plasma membrane. For example, ANO1 is apical in pancreas, salivary gland, and airways, but it is basolateral in mouse colonic epithelia [104,105,106,107]. Cellular location of ANO1 may therefore depend on the cell type, and maybe on the cell function and differentiation. For example, ANO5 is mostly found intracellularly, but it can be also detected in the plasma membrane where it produces a non-selective whole cell current [15,102]. Endogenous and overexpressed ANO10 is typically intracellular, and co-localizes with acetylated tubulin [108,109,110]. However, expression and localization appears tissue dependent and may dependent on the cell cycle. For example, endogenous ANO10 in rapidly proliferating Fisher Rat Thyroid (FTR) cells is mostly intracellular and appears upregulated during mitosis [108] (Figure 1). Once FRT cells form a dense monolayer and stop proliferating in serum free media, some ANO10 moves into the cell membrane and co-localizes with the centrioles [108] (Figure 1A). Non-proliferating cells on permeable supports and in the absence of serum seem to lower expression of ANO10, which is now preferentially expressed close to the centriole and probably in the primary cilium (Figure 1). Expression in the primary cilium has also been observed for ANO1 and ANO6 in renal and retinal pigment epithelial cells [111,112,113]. We may therefore hypothesize a dynamic regulation of expression and localization of anoctamins, depending on proliferation and on the cell cycle (Figure 1B). As discussed below, upregulation of ANO1 is correlated with enhanced proliferation, e.g., in polycystic kidney disease, in many rapidly growing cell lines, as well as in different types of tumors [73,114] (Table 1).

## 3. How Is ANO1 Upregulated during Cell Proliferation and Cancer Development?

**Figure 2:**
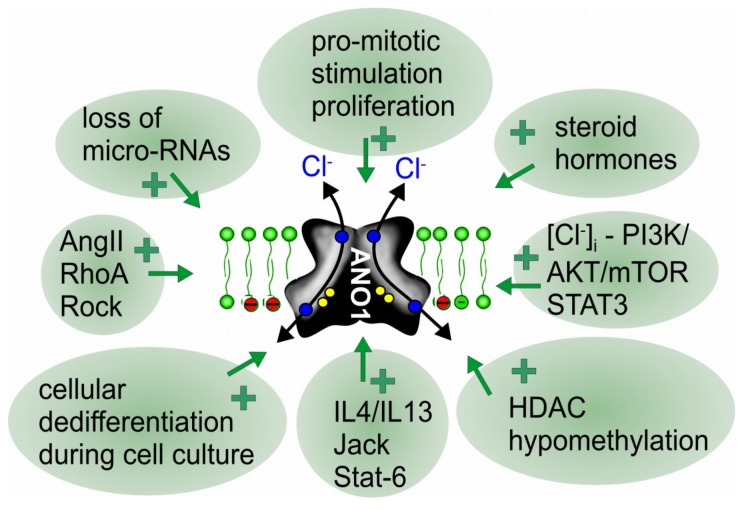
Upregulation and redistribution of ANO1 during proliferation and cancer. Scheme summarizing reported factors and signaling pathways that lead to upregulation of expression of ANO1 and cellular redistribution during proliferation and cancer growth (Table 1). For further details and references, see main text.

Most studies on ANO1 have been performed on cultured cells, particularly in ANO1 overexpressing cells. Under these conditions, ANO1 currents are generally of large size and may show some properties that are different to currents expressed endogenously [95,115]. Although ANO1 is widely expressed and particularly abundant in epithelial cells [15,90,91], we observed that non-proliferating epithelial cells in culture or freshly isolated (non-cultured) cells from airways, kidney and intestine show very little Ca^2+^ activated Cl^−^ currents [116,117,118]. However, ANO1 currents are quickly upregulated once cells have been isolated from the tissue and are maintained in serum-containing media under proliferating conditions. Upregulation of ANO1-currents can be reversed by growing the cells on permeable supports and removing the serum so that cells stop proliferating [11,106,116,117,119,120]. Thus, removing the cells from their physiological environment, cellular reorganization and pro-mitotic stimulation may all contribute to upregulation of ANO1. Moreover, transcriptional stimulation via the IL4/IL13-Jack-STAT3-STAT6 axis, steroid hormones such as testosterone, activation of histone deacetylase (HDCA), promotor hypo- methylation, as well as downregulation of inhibitory micro-RNAs have been shown to upregulate ANO1 expression (reviewed in [73,74,76,121,122] (Figure 2).

## 4. ANO1, Cell Proliferation and Tumor Growth: How Does It Work?

ANO1 was found to increase proliferation in many different tissues [14,26,30,71,72,75,114,123,124,125] (Table 1). Apart from increasing proliferation, additional pro-apoptotic effects of ANO1 have also been reported, based on studies using ANO1-inhibitors. It should be noted, however, that ANO1-inhibitors might exert non-specific effects, when used at higher concentrations. In contrast, inhibition of proliferation by knockout of ANO1-expression or inhibition of ANO1 using low concentrations of ANO1 inhibitors have been shown in a number of studies [73,126,127]. This can also be demonstrated in experiments using nanomolar concentrations of the recently identified potent ANO1 inhibitor niclosamide, which however, has a number of additional anti-cancer effects (c.f. below) [128]. Nevertheless, other ANO1-inhibitors also blocked cell proliferation and cancer growth [30,72,127,129].

**Figure 3:**
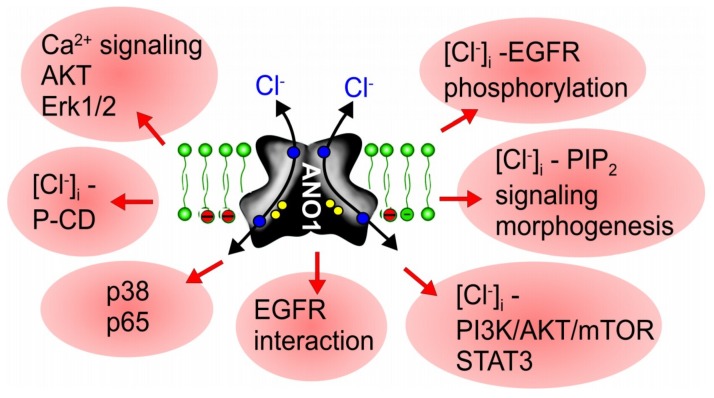
Mechanisms for ANO1-induced cell proliferation and cancer development. Scheme summarizing reported mechanisms for ANO1-induced cell proliferation and development of cancer. All pathways are inhibited by niclosamide and other inhibitors of anoctamins (Table 1). For further details and references, see main text.

Niclosamide is a FDA-approved drug and was shown to inhibit Notch signaling [130], a pathway that is well known to participate in tumorigenesis [131]. In a number of reports, additional antineoplastic mechanisms of niclosamide have been described. Thus, niclosamide was shown to inhibit nuclear factor kappa B (NF-κB), Wnt/ß-catenin signaling, the IL-6-JAK1-STAT3-pathway, GSK-3 and more [132,133,134,135,136,137,138,139,140]. A recent paper suggests cell cycle arrest by niclosamide, through activation of the Let-7d/CDC34 axis [41]. Notably, blockade of notch signaling inhibits goblet cell metaplasia in asthmatic mice, which could be part of the mechanism how niclosamide inhibits mucus production [128,141,142]. Moreover, mucus production is also inhibited by other ANO1 inhibitors, such as niflumic acid (NFA) and CaCCinhAO1, or in ANO1 knockout mice [107,143,144]. Although the antiproliferative effects of niclosamide correspond well to its inhibitory effect on ANO1, this relationship is not well recognized. Niclosamide has been used in a number of preclinical studies and even in clinical trials with prostate and colorectal cancer patients [135,137,145,146,147,148,149]. Taken together, the multiple anti-cancer effects described for the ANO1-inhibitor niclosamide, may correspond to the wide range of pro-cancerous mechanisms by ANO1 (Figure 3).

## 5. ANO5, 6, 7, and 9 in Cancer and Cell Proliferation

Other members of the anoctamin family were also associated with cell proliferation, embryogenesis and cancer growth. ANO5 (TMEM16E) is now known for its role in myoblast proliferation and muscle repair [80,103], while gain of function mutations of ANO5 cause gnathodiaphyseal dysplasia [150]. In a collaborative effort, we identified an essential role of ANO6 for embryogenesis [151]. Similar to ANO5 also ANO6 was also reported to control myoblast proliferation [80]. ANO7 (TMEM16G, NGEP) is a marker for prostate cancer [85,86]. For ANO9 (TMEM16J) an inverse correlation of expression and progression of colorectal cancer was described [89], while it may also promote pancreatic cancer [88]. Interestingly, enhanced phospho-Erk1,2 activity was correlated with the cellular effects of ANO1, 6, and 9, but a possible common mechanism remains obscure [30,80,88]. 

## 6. Anoctamins Control Intracellular Ca^2+^ Levels

Growth hormone receptors signal via Ras/Raf/Erk1,2, PI3K/Akt, and DAG/IP_3_ [152], while intracellular Ca^2+^ signals are essential regulators of cell proliferation [153]. We showed that anoctamins control compartmentalized Ca^2+^ signals ([Ca^2+^]_i_), and therefore proposed this as a major mechanism by which ANO1, and possibly other anoctamins, affect cell proliferation and a number of other cellular properties [108,110,154,155]. It is important to note that ANO1 is homologous to yeast Ist2, known to tether the peripheral cortical endoplasmic reticulum (ER) to the plasma membrane [156]. Gamper and collaborators convincingly showed that the relatively low Ca^2+^ sensitivity of ANO1 at (physiological) negative membrane voltages, requires this mechanism in order to concentrate Ca^2+^ near the plasma membrane and in close proximity of ANO1 [157,158]. ER-localized inositol trisphosphate receptors interact with ANO1 and tether the ER to ANO1 containing plasma membranes [157]. Moreover, IP3-induced Ca^2+^ store release is augmented by ANO1 [110]. Anoctamin-controlled Ca^2+^ compartments could be relevant for expression and activation of Erk1,2 [30,159]. Transient rise in intracellular Ca^2+^ followed by sustained activation of the Ras/Raf/Erk pathway is a central aspect of cell proliferation in many systems [154,160,161,162]. 

**Figure 4:**
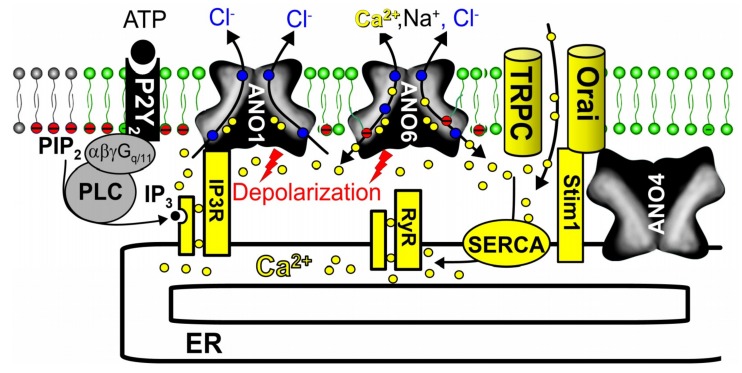
Compartmentalized Ca^2+^ signaling by anoctamins. Scheme illustrating the effects of anoctamins on Ca^2+^ signaling. ANO1 tethers ER Ca^2+^ stores close to the plasma membrane, which leads to improved ATP-induced apical Ca^2+^ signaling. Activation of both ANO1 and ANO6 induce plasma membrane depolarization, supporting release of Ca^2+^ from ER stores via inositol trisphosphate receptors (IP_3_R) and ryanodine receptors (RyR). In addition, Ca^2+^ store content was found to be enhanced by ANO1. ANO6 is permeable for Ca^2+^ and therefore supports Ca^2+^ entry. ANO4 localized in the ER interacts with Orai1 [110]. For further details and references, see main text.

Although detailed mechanisms are currently not fully understood, it is clear that also other anoctamins affect [Ca^2+^]_i_, i.e., basal [Ca^2+^]_i_ as well as receptor mediated Ca^2+^ signals depend on expression of anoctamins [110]. Apart from compartmentalization, protein interaction [163] and membrane depolarization, anoctamins may also contribute to cell proliferation and cell growth by operating as counter-ion channels. Counter ion movement of K^+^ or Cl^−^ over the ER membrane is necessary for charge compensation to allow for efficient Ca^2+^ transport out of the ER via release channels, and for re-uptake of Ca^2+^ into the ER by the sarcoplasmic endoplasmic reticulum Ca^2+^-ATPase (SERCA) [164,165]. Given the Ca^2+^ permeability of some anoctamins, they may also serve as plasma membrane localized Ca^2+^ channels [90,166,167,168] or ER Ca^2+^ leakage channels [103,110,154,169,170,171]. Disturbed intracellular Ca^2+^ signals with changes of cellular properties are detectable in naïve tissues and primary cells from mice with knockout of anoctamins [107,108,110,155,172,173,174] (Figure 4). 

## 7. The Role of Anoctamins in Controlling Intracellular Cl^−^ Concentration, Exocytosis, Organ Growth and Microvesicular Signaling

**Figure 5:**
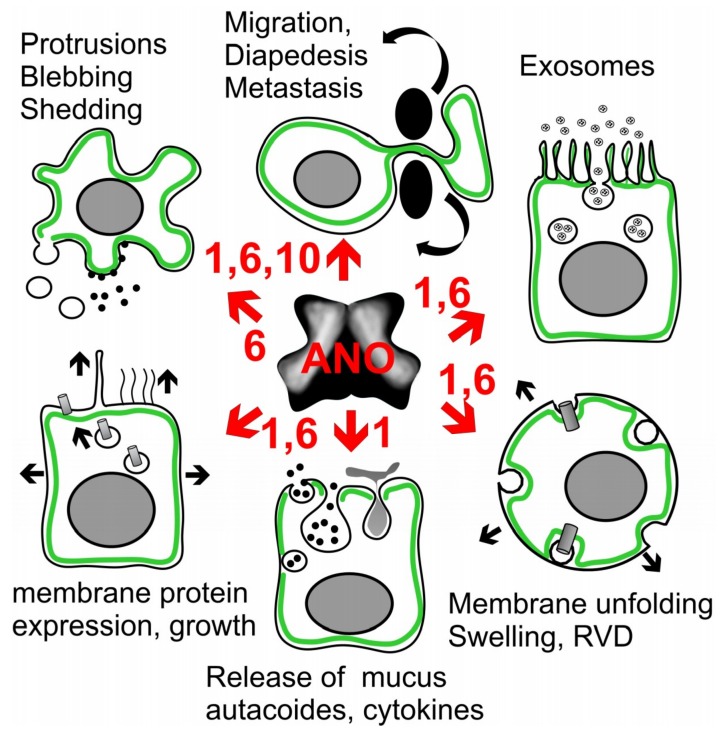
Potential action of anoctamins on exocytosis, growth and microvesicular signaling. ANO1 and ANO6 determine the extent of membrane protrusions and membrane blebbing in macrophages and other cell types, and support cell migration, diapedesis and cancer metastasis. Exosome release and paracrine signaling by epithelial cells is probably anoctamin-dependent. Support of membrane unfolding, cell swelling and subsequent activation of VRAC could be a general property of anoctamins. Mucus secretion and release of inflammatory mediators such as autacoids and cytokines was shown to be ANO1-dependent. Exocytosis leads to enhanced expression of membrane proteins, cell growth, and extensions such as motile cilia and the primary cilium, as proposed for ANO1. For further details and references, see main text.

Recent reports suggest additional mechanisms whereby anoctamins may augment proliferation and cell growth. He and coworkers proposed an interesting concept in which ANO1 controls cytoplasmic Cl^−^ levels that affect phosphoinositide levels in the inner plasma membrane leaflet, such as PtdIns(4,5)P2 in membrane microdomains [175]. Although the proposed concept requires further validation, it could contribute to attenuated purinergic Ca^2+^ signals found in tissues isolated from conditional ANO1 knockout animals or in ANO1 knockout cells (c.f. above). Similar to He et al., we also detected shortened motile cilia in the respiratory epithelium of ANO1-knockout mice, as well as a reduced length of non-motile primary cilia in renal collecting ducts of ANO1 knockout mice. While motile cilia from wt animals measured 6.1 ± 0.4 µm, those from animals with a ANO1-knockout in ciliated epithelial cells had a length of only 3.6 ± 0.4 µm (n = 7). Finally, the data by Ruppersburg and Hartzell convincingly demonstrate the importance of ANO1 for primary ciliogenesis [111]. siRNA-suppression of ANO6 expression and expression of other anoctamins suggested a contribution to basal Cl^−^ conductance [102]. In contrast, overexpression of ANO1 and ANO6 enhanced basal Cl^−^ conductance when analyzed at 37 °C [95]. In contrast to He et al., we found that ongoing activation of ANO1 (or ANO6) by either ionomycin or purinergic stimulation increased intracellular Cl^−^ concentrations in HEK293 and HeLa cells [102]. Nevertheless the proposed concept that intracellular Cl^−^ levels determine vesicular endocytosis/exocytosis, control apical membrane delivery and morphogenesis [175], is interesting and corresponds well to the role of ANO1 in exocytosis and normal renal development detected in recent studies [107,175,176,177] (Figure 5). It is also noteworthy that intracellular Cl^−^ regulation by ANO1 has been shown to participate in transcription of human epidermal growth factor receptor 2, which mediates PI3K/AKT/mTOR and JAK/STAT3 signaling pathways [52].

We observed that animals lacking expression of ANO1 in epithelial cells of airways and intestine accumulate mucus in club (Clara) and goblet cells [107]. We found that ANO1 is essential for secretion of mucus, probably by controlling mucus release from club/goblet cells, and by controlling release of prosecretory cytokines from ciliated cells [107]. IL-13-induced production and secretion of Muc5AC was inhibited by the ANO1 blocker and antiproliferative/anticancer drug, niclosamide. Along the same line, release of IL-8 induced by lipopolysaccharide (LPS) was significantly reduced by knockdown of ANO1. A recent paper by Hilgemann and colleagues demonstrates massive membrane expansion with activation of ANO6, with subsequent membrane shedding [178]. These results are reminiscent to our earlier observations of ANO6-depending blebbing and membrane shedding in macrophages [98]. Taken together, there is now evidence that anoctamins, particularly ANO1 and ANO6, control endolysosomal trafficking [98,107,108,175,179,180], membrane exocytosis, increase in membrane surface area and insertion of proteins into the plasma membrane [155,176,181] (Figure 5). As outlined above, ANO1 and ANO6 control [Ca^2+^]_i_, which is an essential regulator of exocytosis. Thus compartmentalized [Ca^2+^]_i_ close to the plasma membrane is required for docking of exocytic vesicles and granules, respectively. This process requires the so-called Munc13 proteins and the soluble N-ethylmaleimide-sensitive factor-attachment protein receptor machinery [182,183].

Anoctamins were shown to have additional impact on cancer related events that involve plasma membrane function. Both ANO1 and ANO6 support cell migration and metastasis [14,26,30,55,61,73,184]. Endogenous ANO6 expressed in macrophages, or ANO6 overexpressed in HEK293 cells, induced massive membrane blebbing when activated by the P2X_7_-agonist ATP [98]. ANO6 also supported apoptosis, movement, and formation of protrusions, as well as phagocytic activity and bacterial killing by macrophages [98,108]. Importantly, phosphatidylserine exposure by ANO6 is required for the function of ADAM17 and ADAM10, both members of the family of cell bound disintegrin and metalloproteases. These enzymes regulate a plethora of biological functions, including proliferation and cell death [185,186,187]. The role of ANO1 for organ development, cell growth and extension of motile cilia and primary cilia has been discussed above. This is in line with its contribution to exocytosis and release of mucus or cytokines [107,155,176,188,189]. Moreover, other papers report a function of ANO6 [178] and ANO1 [190] for the release of microvesicles and exosomes, which could represent a paracrine control of neighbor cells in airways and intestine [107,191,192,193,194]. Notably, ANO1 is excreted in human urinary exosomes [195]. Interestingly, for both tissue repair [196] and necroptotic cell death [197] a role of the endosomal-sorting complex required for transport (ESCRT) has been described. Correspondingly, repair of muscle membrane requires ANO5 [103,198], while ANO6 has a role in necroptotic cell death [197,199]. Finally, our previous work suggests a role of ANO1, ANO6 and ANO10 in both membrane swelling and volume regulation by regulatory volume decrease, which is related to membrane unfolding and phospholipid metabolism [109,176,200] (Figure 5). This will be described in more detail below.

## 8. ANO1 Is Upregulated during Inflammation

The current data suggest that upregulated ANO1 in rapidly growing cells and tumors, supports proliferation, while expression in differentiated non-proliferating cells is generally much lower and may enable cells to perform specific tasks such as signaling, contraction, or secretion of electrolytes and mucus. Proliferation and inflammation/hypoxia are intimately connected through multiple signaling pathways including JACK/STAT [201,202,203]. Thus, it is not surprising that ANO1 is strongly upregulated during inflammation, which enabled its molecular identification as CaCC [6,204]. ANO1 is strongly upregulated in inflammatory airway diseases such as CF, COPD and asthma, which parallels goblet cell metaplasia and mucus hypersecretion [143,181,205]. It is also upregulated during bacterial inflammation [206]. Upregulation of TMEM16A is predominant in mucus producing cells and to a lesser degree in ciliated airway epithelial cells [107,155,205,207]. ANO1 may participate in the transition from inflammation to proliferation, which explains its strong impact in wound healing and tissue repair [14,103,208].

## 9. Relationship of Anoctamins to the Tumor Associated Cl^−^ Channel VRAC

All living cells are able to maintain a constant cell volume. According to a general concept, regulatory volume decrease (RVD) prevents cell swelling and necrotic cell death, while regulatory volume increase (RVI) prevents cell shrinkage and apoptotic cell death [209,210]. The volume regulated or swelling activated anion channel (VRAC) is activated during RVD. Excessive activation of VRAC may support apoptotic cell death, while its upregulation leads to cellular resistance towards anti-cancer drugs [209,211,212,213,214,215,216,217]. Recent experiments suggest that ANO1 and ANO6 also contribute to volume activated whole cell currents, which may indicate a possible functional link between anoctamins and VRAC [123,176,200,218]. Although broadly expressed, there has been a long controversy concerning the molecular identity of VRAC, which was finally solved in 2014 [219,220,221]. Structural analysis by cryo-EM demonstrated a hexameric assembly of LRRC8 subunits, which form a typical ion channel with a central pore, structurally related to the connexin family of channels [222,223,224]. Conserved charged amino acid residues at the extracellular domain determine the permeability towards anions and other osmolytes. Two structurally different populations of VRAC have been shown by Kasuya et al., corresponding to a compact and a relaxed conformation. These conformations may correlate to closed and open states of the channel [224].

Although rather abundant, LRRC8/VRAC may not be essential for RVD and thus cells may be able to control their cell volume in the absence of VRAC [225,226,227]. Lack of functional VRAC leads to increased prenatal and postnatal mortality, growth retardation, and multiple tissue abnormalities, including abnormal function of B- and T-cells [228,229,230,231]. Additional, LRRC8-independent and cell-specific mechanisms may exist that enable RVD. These mechanisms comprise other Cl^−^ channels such CFTR, bestrophin, and anoctamins, as well as electroneutral KCl co-transporter [218,225,226,227]. An inverse relationship exists between the magnitudes of VRAC and of Ca^2+^ activated ANO1 Cl^−^ currents: With increased activation of CaCC, VRAC decreases and vice versa [232,233]. We could not activate ANO1 after maximal activation of VRAC [123], while Zholos et al showed a reduced probability for activation of VRAC after activation of CaCC [233]. A loss of expression of LRRC8A not only inhibited VRAC, but also attenuated Ca^2+^ activated Cl^−^ currents. Vice versa, overexpression of LRRC8A enhanced Ca^2+^ activated Cl^−^ currents. Because LRRC8A and ANO1 could be co-immunoprecipitated, a co-localization of both anion channels is proposed, with membrane insertion of LRRC8A being supported by ANO1 [176]. Apart from ANO1 and ANO6, also VRAC is blocked by niclosamide [95]. Because VRAC induces resistance towards cisplatin and other anticancer drugs and leads to metastasis and bad patient outcome, inhibition of VRAC may be another mechanism how niclosamide inhibits growth of cancer [215,234,235].

Taken together, a functional relationship exists between VRAC and ANO1, possibly because activation of both channels involves release of Ca^2+^ from the ER-store [200,236]. As VRAC controls survival of cells, the functional crosstalk with ANO1 is highly relevant for tumor biology [215,234,236].

## 10. Role of Anoctamins in Cell Death

Sustained large increase in intracellular Ca^2+^ can lead to senescence or cell death [237,238]. We showed earlier that P2X_7_-mediated increase of intracellular Ca^2+^ leads to cell death of macrophages and lymphocytes expressing endogenous ANO6, and of HEK293 cells overexpressing ANO6 [98,239]. ANO6 is a component of the so-called outwardly rectifying Cl^−^ channel ORCC or ICOR, and has a role in cell shrinkage and programmed cell death [218,239,240,241]. Expression of ANO6 is dominant in the surface epithelium of large intestine, were aged enterocytes die and dead cells are exfoliated. ANO6 is not found in intestinal crypts, where enterocytes are produced from stem cells [239]. TUNEL assays performed in mouse intestinal epithelium lacking ANO6 expression, unmask reduced cell death, when compared to wt mice. In addition, ANO10 is important for spontaneous and TNFα-induced cell death in mouse intestinal epithelium, peritoneal macrophages, and THP1 macrophages [108]. Moreover, knockdown of ANO6 impaired apoptosis and formation of cyst lumen in 3D cultures of MDCK renal cysts. ANO6 is normally expressed in apoptotic cells within the center of growing cysts formed by MDCK cells and human polycystic kidneys [113].

Although many studies demonstrated the pro-proliferative role of ANO1, a pro-apoptotic function of ANO1 has also been reported [125,242,243]. Almaca et all showed that activation of ANO1 can lead to apoptotic cell shrinkage [123]. Interestingly, the genes for ANO1 and for apoptosis associated Fas associated via death domain (FADD), are located on a common amplicon located on chromosome 11q13. Surprisingly, both proteins were associated with better survival of HNSCC patients [38]. In contrast to ANO1, which is unable to scramble membrane phospholipids, ANO6-induced cell death is probably related to its ability to scramble membrane phospholipid [99]. Interestingly, we found that expression of ANO1 enhanced ionomycin-induced scrambling performed by endogenous ANO6 in HEK293 cells. This may point to a synergism between both anoctamins, which has also been detected earlier for Cl^−^ currents produced by both proteins [244]. 

Meanwhile, a number of independent regulated cell death pathways have been identified [245]. Initially, ANO6 has been reported in the context of apoptosis, but is now shown to be activated also during necroptosis, pyroptosis and ferroptosis [95,199,246,247,248]. Thus, activation of anoctamins, particularly of ANO6, might be a possibility to induce cell death in cancer cells.

## 11. Activation of Anoctamins and Ferroptotic Cell Death in Cancer

Pro-apoptotic Cl^−^ currents have been activated in cells overexpressing ANO1, ANO6, ANO9 and ANO10 [95,123,227]. A potent activator of anoctamins is the bee venom melittin, which stimulates phospholipase A2 (PLA_2_) [95,200]. Noteworthy, melittin has been widely used as anti-cancer therapy, and PLA_2_-dependent activation of metalloproteinase is essential for this effect [249,250,251].

Anoctamins are also activated through reactive oxygen species and by lipid peroxidation. This may lead to inflammation and proliferation, ion secretion and ferroptosis, depending on the cell type, the anoctamin paralogue being activated, and the strength of peroxidation [95,118,247,252,253]. Ferroptosis is induced by accumulation of intracellular iron, and is distinct from apoptosis, necrosis, and other forms of regulated cell death [254]. Ferroptosis is triggered by an increase in reactive oxygen species (ROS) and an overwhelming lipid peroxidation that ultimately leads to cell death by disintegration of the plasma membrane. Experimentally lipid peroxidation is also induced by erastin-inhibition of cysteine import through the transporter system X_c_^−^. This leads to depletion of glutathione (GSH) and inactivation of the phospholipid peroxidase glutathione peroxidase 4 (GPX4). In addition, GPX4 can be directly inhibited by RSL3 [255]. 

Cell death can be induced in cancer cells by activation of ANO6 through melittin-induced PLA_2_ or through lipid peroxidation [95]. This may suggest a new potential therapeutic approach to inhibit growth of cancer [95,247]. Lipid peroxidation and ferroptosis-induced cell death was proposed earlier as a mechanism to destroy cancer cells [256]. However, the ROS buffer capacity is typically quite high in cancer cells, which will antagonize lipid peroxidation [257]. ROS levels could be enhanced to exceed the antioxidant defense of cancer cells [258]. A number of preclinical studies were performed using small molecules to inhibit cellular glutathione antioxidant activity [259,260,261,262]. Tumor cell lines that were killed by the ANO6-activator melittin were also driven into ferroptosis by erastin and RSL3. Thus ANO1 and ANO6 were shown to be activated during ferroptotic cell death [118,247]. 

## 12. Conclusions

Proteins of the anoctamin/TMEM16 family scramble membrane phospholipids and operate as Cl^−^ and cation-permeable channels. They demonstrate impressive effects on basic cell properties, and support both cell proliferation and regulated cell death. Clearly more work is required to be able to define the cellular functions of anoctamins, and their role in proliferation and cancer development. Despite the plethora of underlying cell specific signaling pathways, it will be interesting to learn whether common mechanisms exist for the cellular effects induced by anoctamins, such as enhanced intracellular Ca^2+^ signaling. Blocking ANO1 appears feasible to interfere with cancer growth. 

**Figure 6:**
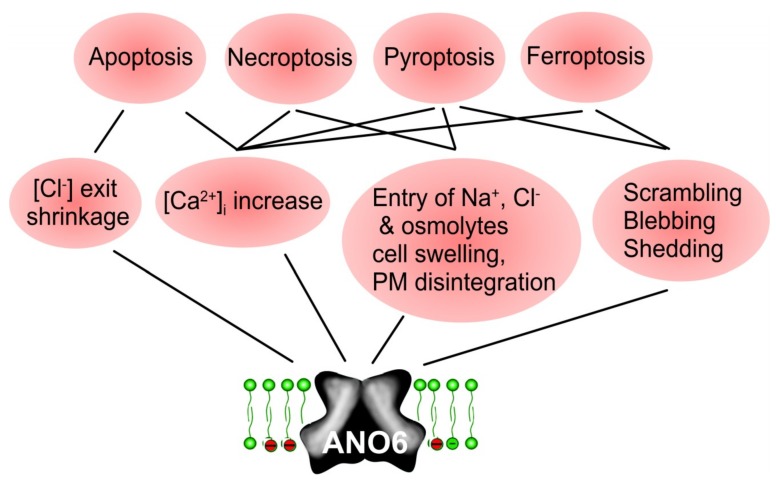
ANO6-induced cell death. Scheme summarizing the contribution of ANO6 to different regulated cell death pathways such as apoptosis, necroptosis, pyroptosis, and ferroptosis. Anoctamins may contribute to regulated cell death by cell shrinkage (apoptosis), increase in compartmentalized intracellular Ca^2+^ (all cell death pathways), or cell swelling, scrambling, blebbing, and membrane disintegration (ferroptosis, pyroptosis). For further details and references, see main text.

In contrast to the pro-proliferative effect of ANO1, ANO6 seem to contribute to different types of regulated cell death (Figure 6). Activation of ANO6 may cause swelling or shrinkage of cells, and does increase in intracellular Ca^2+^, phospholipid scrambling, membrane blebbing and membrane shedding. It may all contribute to ANO6-induced cell death. Thus, direct activation of ANO6 may be a promising new strategy to induce cell death in cancer cells.

## Figures and Tables

**Table 1:** Anoctamins in Cancer and Proliferation.

Anoctamin Paralogue	References
**Anoctamin 1, TMEM16A**	
GIST, squamous carcinoma, head and neck cancer	[12,13,14,17,18,19,20,21,22,23,24,25,26,27,28,29,30,31,32,33,34,35,36,37,38,39,40,41]
Pancreatic cancer	[42,43,44]
Prostate cancer	[45,46,47]
Breast cancer	[48,49,50,51,52,53]
Colorectal carcinoma	[54,55]
Gastric cancer	[56,57]
Glioma, Glioblastoma	[58,59]
Esophageal cancer	[60]
Lung cancer	[61,62,63]
Hepatocellular carcinoma	[64]
Ovarian cancer	
Liposarcoma	[65]
Leimyosarcoma	[66]
Salivary gland cancer	[67]
Chondroblastoma	[68]
General role in cancer and proliferation	[14,69,70,71,72,73,74,75,76]
**Anoctamin 5, TMEM16E**	
Colorectal cancer	[77,78]
Thyroid cancer	[79]
**Anoctamin 6, TMEM16F**	
Myoblast proliferation	[80]
**Anoctamin 7, TMEM16G**	
Prostate cancer	[81,82,83,84,85,86]
Breast cancer	[87]
**Anoctamin 9, TMEM16J**	
Pancreatic cancer	[88]
Colorectal carcinoma	[89]
